# Analytical Model of Temperature-Induced Deformation for Tunable Thermal Expansion Metamaterial

**DOI:** 10.3390/ma18030532

**Published:** 2025-01-24

**Authors:** Ling Xiao, Yaxin Yao, Shuai Chen, Mengting Lai, Guanghong Zhu

**Affiliations:** 1 Department of Mechanics, Xi’an University of Science and Technology, Xi’an 710054, China; yaoyaxin219@163.com (Y.Y.); 18220528990@163.com (M.L.); zhuguanghong@xust.edu.cn (G.Z.); 2 National Key Laboratory of Science and Technology on Advanced Composites in Special Environments, Harbin Institute of Technology, Harbin 150001, China

**Keywords:** tunable thermal expansion metamaterial (TTEM), virtual work principle, temperature-induced deformation, coverage ratio

## Abstract

Tunable thermal expansion metamaterials exhibit superior shock absorption performance in the field of high-precision equipment, but the applications are currently restricted by the unclear quantitative relationship of temperature-induced deformation. Herein, this work leverages the virtual work principle and the deformation geometric relationship to establish a generic temperature-induced deformation control model for bi-materials by utilizing the key variable coverage ratio under the condition of no deformation in the vertical direction. The feasible region regarding flexibility for the internal serpentine unit and lattice structure with different coverage ratios is given. The combination of the finite element and experimental methods is adopted to examine temperature-induced deformation, which presents tunable thermal expansion performances associated with the coverage ratio and temperature. This work, based on the established deformation coordination relationship of dual-material temperature-sensitive metamaterials, achieves temperature-induced deformation control and provides a reference for structural design adaptable in various working conditions such as vibration isolation and vibration reduction in complex engineering such as aerospace and so on. By strategically designing the coverage of the two structures within the specified range to maintain equivalent flexibility, the ultimate deformation of the serpentine unit is reduced by one-half due to deformation induced by temperature variations.

## 1. Introduction

The increasing demand for thermal stability of systems under extreme environments, has led to metamaterials with tunable thermal expansion, negative stiffness [1,2], and extraordinary physical properties mainly through structural design becoming a research hotspot in the field of new materials [3]. Dual requirements for thermal and mechanical properties have motivated the design of stretch-dominated and bending-dominated [3,4,5] bi-material lattices induced by thermal exposure as an effective compensation for volume change.

Currently, many structures such as the slender straight or curved composite beam [6,7,8], the honeycomb structures including the simple honeycomb structure [9], the novel hybrid honeycomb structure by merging two hexagonal lattices [10], the lightweight 3D honeycomb structure by integrating concave double arrows with dual-material triangles [11], the 3D star-shaped honeycomb structure [12], and the 3D dual-material triangular cavity honeycomb structure [13] have been designed. However, the coefficient of thermal expansion (CTE) and negative stiffness are affected by geometric parameters, positional parameters, and material type. It is worth noting that a tunable metamaterial joint inspired by the molecular structure of carbon nanotubes has been proposed [14]. This joint can effectively absorb impact energy, reducing plastic deformation of material components. This further demonstrates the crucial influence of microstructural design on macroscopic mechanical performance and offers innovative perspectives and reference points for the design of the bi-material structure in the present research. Particularly, stress concentration on the triangular or star-shaped honeycomb structure is more likely to occur and has a larger numerical value.

Subsequently, the double-layer design based on lattice and mesh structures has gradually attracted widespread attention. Horseshoe metamaterials can achieve a notable negative Poisson’s ratio and adjustable “J-shaped” stress–strain curves [15]. Significant progress has been made in the structural design of microstructures with geometric forms such as horseshoe [16,17,18], serpentine [19], and triangular [20] units. Subsequently, soft network materials with rotatable structural lattice nodes are designed by adding ring or disk-shaped structures to the horseshoe microstructure, which provide high stretchability while maintaining high strength [21]. Serpentine metamaterials exhibit high stretchability and temperature adaptability, and the strain and stiffness can be regulated by adjusting the width of the serpentine belt, arc radius, arc length, and arm length [19]. Metamaterials made up of triangular micro-units possess high stiffness and strength [22]. They are composed of trapezoidal micro-units that can achieve a broader range of thermal expansion tunability from positive to negative values in two orthogonal directions [20]. Four-dimensional printed intelligent lattice metamaterials can regulate the stiffness, energy absorption, and vibration damping effects by their altering structural parameters. However, their performance is significantly affected by temperature, where excessive temperatures lead to a decline in material strength, while excessively low temperatures impact energy absorption capability [23]. Furthermore, bifunctional metamaterials with different combinations of Poisson’s ratio (PR) and coefficient of thermal expansion (CTE) can selectively deform under mechanical loading and cause dimensional changes under thermal loading, but the specific size cannot be estimated [24].

Furthermore, to meet the performance changes in materials in thermal environments and the specific requirements for thermal expansion behavior of engineering structures such as large space telescopes or antennas, it is necessary to fully consider the impact of temperature on the mechanical properties of materials from a theoretical perspective, which traditional materials cannot achieve. Recently, a thermally sensitive metamaterial assembled from highly sensitive thermostatic metal strips was produced, which exhibits significant shape transformation capabilities during the process of thermal energy conversion. The metamaterial is capable of achieving a design strain of 70% to 80% in just 5 s of heating, and reaches a thermal strain of approximately 30%, which is significantly higher than that of other bi-metallic metamaterials [25]. Based on the Euler beam theory, the temperature-dependent models for the thermal expansion and stiffness of metamaterials that consider the stability control and natural frequency enhancement factors have been established. It can accurately predict expansion-induced deformation and then improve structure reliability [20]. Furthermore, studies have investigated the photothermally activated energy transfer effects in single-walled carbon nanotube thin films using a vectorial two-wave mixing technique. These investigations have revealed that quantum and nonlinear optical phenomena, with varying degrees of involvement, lead to significant changes in the equivalent incident energy produced by the optical interaction of a single beam and two-wave mixing in terms of thermal conduction. This research underscores the importance of microstructural design on the macroscopic mechanical properties of materials and offers new insights and directions for the design of bi-material structures in this study [26]. Three-dimensional printed programmable horseshoe lattice structures based on a phase-evolution model may exhibit incomplete shape recovery due to non-uniform heating, which limits its application in areas requiring precise shape memory and uniform deformation recovery [27]. Two-dimensional expansion coefficients and stiffness models based on energy and stiffness matrix methods can be used to design metamaterials that can adapt to temperature changes and possess programmable CTE and stiffness [21]. Nevertheless, the deformation coordination relationship of metamaterials designed with double-layer assembly strategies in practical applications still needs further clarification. Additionally, there is insufficient guidance on how to precisely adjust these parameters in actual engineering to achieve non-deformation in specific directions.

According to the above information, the related research on this material mainly focuses on the simulation and experimentation of its thermal expansion behavior by changing the material’s parameters. There is no complete design theory to calculate the temperature-induced deformation of bi-, tri-, or more metamaterial. Therefore, it is necessary to explore the intrinsic deformation relationships for a better optimization design.

Given the above requirements, this paper uses the bi-material serpentine unit as an example to conduct an in-depth analysis of its intrinsic deformation relationships. The displacement functions for the bi-material serpentine unit are established based on the principle of virtual work under different loads, respectively. The deformation coordination relationship between the internal serpentine unit and external lattice structure is obtained under the conditions of no deformation in the vertical direction. The feasible region with different coverage ratios is given and verified utilizing the finite element method and experimentation, which can be used for the structural design of temperature-sensitive bi-metamaterials.

## 2. Models and Methods

### 2.1. Geometry of the TTEM

**Figure 1:**
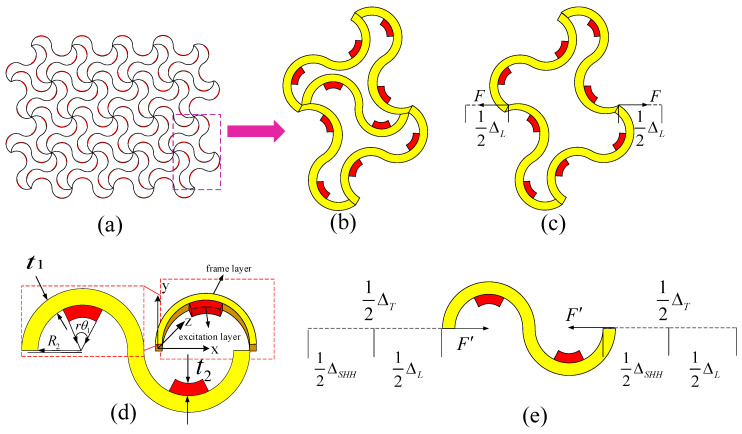
Structures and parameters of the tunable thermal expansion metamaterial. (**a**) Schematic illustration of the tunable thermal expansion metamaterial; (**b**) a 2D unit cell; (**c**) thermal expansion deformation of the lattice structure; (**d**) bi-material serpentine unit; and (**e**) thermal expansion deformation of the serpentine unit.

The bi-material serpentine unit in TTEM is shown in Figure 1a,b, where it evolved from the horseshoe-shaped microstructure (yellow part). The elementary unit for the TTEM, as shown in Figure 1d, is an internal serpentine unit which is superimposed by an internal red actuation layer and an external yellow frame layer. There is a difference in the thermal expansion between the inner red actuation layer and the outer yellow frame layer. In this structure, the thermal expansion coefficient of the inner red actuation layer is higher than that of the outer yellow frame layer. Under the conditions of maintaining the longitudinal displacement unchanged, the material is capable of free deformation in the cross-sectional plane under the influence of temperature. Given that the length in the Z-direction is relatively longer compared to the X-direction, the deformation in the Z-direction is relatively small compared to the initial length. Based on this analysis, this study neglects the deformation effects in the Z-direction. When a temperature change ΔT occurs, due to the difference in the coefficients of thermal expansion between the two material layers, the semicircular double-layer beam undergoes local bending or rotation. According to the Euler–Bernoulli beam theory, the relationship between the linear strain and temperature has been analyzed [28].

(1) $$\varepsilon_{T}=\alpha_{meta}\Delta T=\beta (E,E_{2},\frac{t_{1}}{R_{2}},\frac{t_{2}}{R_{2}})f(\theta_{1},r)(\alpha_{2}-\alpha_{1})\Delta T$$

where

(2) $$\beta (E,E_{2},\frac{t_{1}}{R_{2}},\frac{t_{2}}{R_{2}})=\frac{6(\frac{t_{1}}{R_{2}}+\frac{t_{2}}{R_{2}})}{\frac{Et_{1}}{E_{2}t_{2}}{\left(\frac{t_{1}}{R_{2}}\right)}^{2}+\frac{E_{2}t_{2}}{Et_{1}}{\left(\frac{t_{2}}{R_{2}}\right)}^{2}+4{\left(\frac{t_{1}}{R_{2}}\right)}^{2}+4{\left(\frac{t_{2}}{R_{2}}\right)}^{2}+6\frac{t_{1}}{R_{2}}\frac{t_{2}}{R_{2}}}$$

(3) $$f(\theta_{1},r)=\frac{2\sin(r\theta_{1}/2)-r\theta_{1}\cos(\theta_{1}/2)}{2\sin(\theta_{1}/2)}$$

where $t_{1}$, E, $\alpha_{1}$, $\theta_{1}$, and $R_{2}$ represent the wall thickness, elastic modulus, coefficient of thermal expansion, angle, and inner diameter of the frame layer; $t_{2}$, $E_{2}$, and $\alpha_{2}$ represent the wall thickness, elastic modulus, and thermal expansion coefficient of the excitation layer; r(0≤r≤1) is the ratio of the incentive layer material to the frame layer. As shown in Equations (1)–(3), the effective thermal expansion coefficient of metamaterials $\alpha_{meta}$ is highly dependent on the geometric parameters $t_{1}/R_{2}$, $t_{2}/R_{2}$, r, and $\theta_{1}$. Thus, the suitable equivalent thermal expansion coefficient can be obtained by adjusting the geometric parameters, and then the temperature-induced deformation can be restricted according to actual needs.

### 2.2. Displacement Functions for the Bi-Material Serpentine Unit

**Figure 2:**
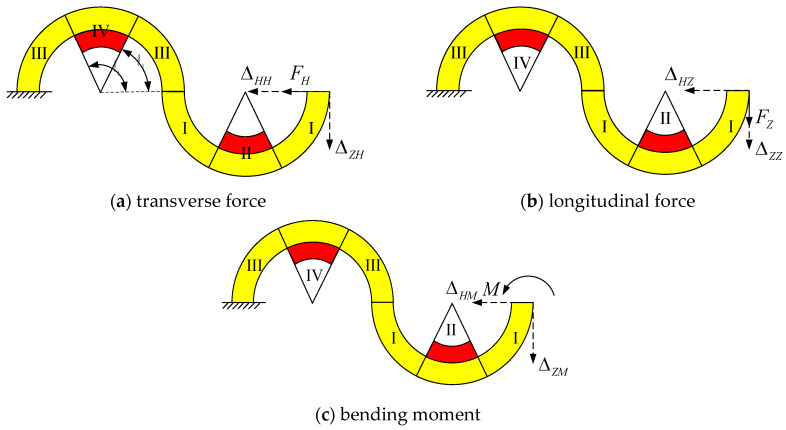
Mechanical models for the serpentine unit.

The tunable thermal expansion metamaterials deform with temperature changes and are subjected to complex forces. In this section, a bi-material serpentine unit is considered for analysis which is the smallest structure of the given material. According to the principle of simplification of the force system, the complex force system can be simplified into three basic loads including transverse force, longitudinal force, and bending moment. In Figure 2, the bi-material serpentine unit is divided into I–IV regions. $\theta_{0}$ and $\theta_{t}$ are the start and end angles of the actuation layer, respectively. Among them, the solid line represents the load, and the dashed line represents the displacement. When the serpentine unit is subjected to a transverse force $F_{H}$, it produces transverse deformation $\Delta_{HH}$ and longitudinal deformation $\Delta_{ZH}$, as shown in Figure 2a. When the serpentine unit is subjected to a longitudinal force $F_{Z}$ or bending moment M, deformations are generated, as shown in Figure 2b,c.

Based on the principles of virtual work, the displacement of the free end under different loads is calculated by Equation (4).

(4) $$\begin{array}{ll} \Delta & =\Delta_{I}+\Delta_{II}+\Delta_{III}+\Delta_{IV}\\ & =(\sum \int \frac{\bar{M_{1}}M_{1}}{ER_{C}S}ds+\sum \int \frac{k\bar{F_{S1}}F_{S1}}{GA}ds+\sum \int \frac{\bar{F_{N1}}F_{N1}}{EA}ds+\sum \int \frac{\bar{F_{N1}}M_{1}}{EAR_{C}}ds\\ & +\sum \int \frac{\bar{M_{1}}F_{N1}}{EAR_{C}}ds)+(\sum \int \frac{\bar{M_{1}}M_{1}}{E_{d}R_{Cd}S_{d}}ds+\sum \int \frac{k\bar{F_{S1}}F_{S1}}{G_{d}A_{d}}ds+\sum \int \frac{\bar{F_{N1}}F_{N1}}{E_{d}A_{d}}ds\\ & +\sum \int \frac{\bar{F_{N1}}M_{1}}{E_{d}R_{Cd}}ds+\sum \int \frac{\bar{M_{1}}F_{N1}}{E_{d}A_{d}R_{Cd}}ds)+(\sum \int \frac{\bar{M_{2}}M_{2}}{ER_{C}S}ds+\sum \int \frac{k\bar{F_{S2}}F_{S2}}{GA}ds\\ & +\sum \int \frac{\bar{F_{N2}}F_{N2}}{EA}ds+\sum \int \frac{\bar{F_{N2}}M_{2}}{EAR_{C}}ds+\sum \int \frac{\bar{M_{2}}F_{N2}}{EAR_{C}}ds)+(\sum \int \frac{\bar{M_{2}}M_{2}}{E_{d}R_{Cd}S_{d}}ds\\ & +\sum \int \frac{k\bar{F_{S2}}F_{S2}}{G_{d}A_{d}}ds+\sum \int \frac{\bar{F_{N2}}F_{N2}}{E_{d}A_{d}}ds+\sum \int \frac{\bar{F_{N2}}M_{2}}{E_{d}R_{Cd}}ds+\sum \int \frac{\bar{M_{2}}F_{N2}}{E_{d}A_{d}R_{Cd}}ds) \end{array}$$

where $M_{1}$, $F_{N1}$ and $F_{S1}$ are the bending moment, axial force, and shear force of any section on the right segment of the serpentine unit when the free end is subjected to an external load, while ${\bar{M}}_{1}$, ${\bar{F}}_{N1}$ and ${\bar{F}}_{S1}$ are the corresponding internal forces of the same section. $M_{2}$, $F_{N2}$ and $F_{S2}$ are the corresponding forces of any section on the left segment, while ${\bar{M}}_{2}$, ${\bar{F}}_{N2}$ and ${\bar{F}}_{S2}$ are the corresponding internal forces of the same section. G, E, $G_{2}$, and $E_{2}$ are the shear and elastic modulus of the frame layer material and the excitation layer material, respectively. $G_{d}=(AG+A_{2}G_{2})/(A+A_{2})$ and $E_{d}=(AE+A_{2}E_{2})/(A+A_{2})$ represent the partial shear strength and elastic modulus of the serpentine unit, k, A, $S=Ay=A\left[R_{C}-t_{1}/\ln(R_{1}/R_{2})\right]$, $R_{c}$, and $A_{2}$ are the correction factors for the uneven distribution of shear stress along the section, the cross-sectional area of the frame layer, the area moment of the frame layer section to the central axis, the radius of the centroid of the frame layer section, and the cross-sectional area of the excitation layer, respectively. $A_{d}=A+A_{2}$ is the composite cross-sectional area. $S_{d}=(A+A_{2})\left\{R_{Cd}-(t_{1}+t_{2})/\left[\ln(R_{1}/(R_{2}-t_{2}))\right]\right\}$ is the area moment of the composite cross-section to the composite central axis. $R_{Cd}=R_{C}-t_{2}/2$ is the radius of the composite cross-section centroid.

**Figure 3:**
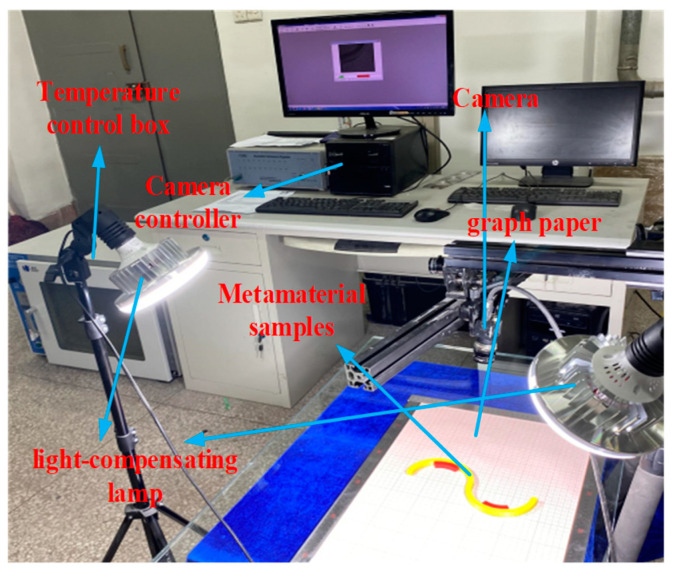
Heating and testing equipment.

It is known that both the transverse and the longitudinal displacements of the free end are produced when the serpentine unit is subjected to the transverse force shown in Figure 3. Under the action of the transverse force, the internal force equation of each part for the serpentine unit can be obtained by the section method.

(5) $$\begin{array}{l} right\;section:M_{1}=F_{H}R_{C}\sin\theta ,F_{S1}=-F_{H}\cos\theta ,F_{N1}=-F_{H}\sin\theta \\ left\;section:M_{2}=-F_{H}R_{C}\sin\theta ,F_{S2}=F_{H}\cos\theta ,F_{N2}=-F_{H}\sin\theta \end{array}$$

The transverse and longitudinal displacements $\Delta_{HH}$, $\Delta_{ZH}$ can be obtained by the section method under the action of virtual transverse and longitudinal forces. The internal forces of each part for the serpentine unit under the virtual transverse force is as follows:

(6) $$\begin{array}{l} right\;section:{\bar{M}}_{1}=R_{C}\sin\theta ,{\bar{F}}_{S1}=-\cos\theta ,{\bar{F}}_{N1}=-\sin\theta \\ \;left\;section:{\bar{M}}_{2}=-R_{C}\sin\theta ,{\bar{F}}_{S2}=\cos\theta ,{\bar{F}}_{N2}=-\sin\theta \end{array}$$

Substituting Equations (5) and (6) into Equation (4), the transverse displacement $\Delta_{HH}$ is as follows:

(7) $$\Delta_{HH}=K_{HH}F_{H}$$

The internal forces of each part for the serpentine unit under the virtual longitudinal force is as follows:

(8) $$\begin{array}{l} right\;section:{\bar{F}}_{N1}=-\cos\theta ,{\bar{F}}_{S1}=\sin\theta ,{\bar{M}}_{1}=R_{C}(\cos\theta -1)\\ left\;section:{\bar{F}}_{N2}=\cos\theta ,{\bar{F}}_{S2}=\sin\theta ,{\bar{M}}_{2}=R_{C}(\cos\theta -3) \end{array}$$

Substituting Equations (5) and (8) into Equation (4), the longitudinal displacement $\Delta_{ZH}$ is as follows:

(9) $$\Delta_{ZH}=K_{ZH}F_{H}$$

According to the above analysis, the transverse displacement flexibility $K_{HH}$ and longitudinal displacement flexibility $K_{ZH}$ of the serpentine unit under transverse force are as follows:

(10) $$\begin{array}{ll} K_{HH} & =(\frac{R_{C}^{2}}{ES}-\frac{R_{C}}{EA})(\int_{0}^{\theta_{0}}\sin^{2}\theta d\theta +\int_{\theta_{t}}^{\pi }\sin^{2}\theta d\theta )+\frac{kR_{C}}{GA}(\int_{0}^{\theta_{0}}\cos^{2}\theta d\theta +\int_{\theta_{t}}^{\pi }\cos^{2}\theta d\theta )+\\ & (\frac{R_{Cd}^{2}}{E_{d}S_{d}}-\frac{R_{Cd}}{E_{d}A_{d}})\int_{\theta_{0}}^{\theta_{t}}\sin^{2}\theta d\theta +\frac{kR_{Cd}}{G_{d}A_{d}}\int_{\theta_{0}}^{\theta_{t}}\cos^{2}\theta d\theta +\\ & (\frac{R_{C}^{2}}{ES}+\frac{3R_{C}}{EA})(\int_{0}^{\theta_{0}}\sin^{2}\theta d\theta +\int_{\theta_{t}}^{\pi }\sin^{2}\theta d\theta )+\frac{kR_{C}}{GA}(\int_{0}^{\theta_{0}}\cos^{2}\theta d\theta +\int_{\theta_{t}}^{\pi }\cos^{2}\theta d\theta )+\\ & (\frac{R_{Cd}^{2}}{E_{d}S_{d}}+\frac{3R_{Cd}}{E_{d}A_{d}})\int_{\theta_{0}}^{\theta_{t}}\sin^{2}\theta d\theta +\frac{kR_{Cd}}{G_{d}A_{d}}\int_{\theta_{0}}^{\theta_{t}}\cos^{2}\theta d\theta \end{array}$$

(11) $$\begin{array}{ll} K_{ZH} & =(\frac{R_{C}^{2}}{ES}-\frac{R_{C}}{EA})(\int_{0}^{\theta_{0}}\sin\theta (\cos\theta -1)d\theta +\int_{\theta_{t}}^{\pi }\sin\theta (\cos\theta -1)d\theta )+\\ & \frac{kR_{C}}{GA}(\int_{0}^{\theta_{0}}-\sin\theta \cos\theta d\theta +\int_{\theta_{t}}^{\pi }-\sin\theta \cos\theta d\theta )+\\ & (\frac{R_{Cd}^{2}}{E_{d}S_{d}}-\frac{R_{Cd}}{E_{d}A_{d}})\int_{\theta_{0}}^{\theta_{t}}\sin\theta (\cos\theta -1)d\theta +\frac{kR_{Cd}}{G_{d}A_{d}}\int_{\theta_{0}}^{\theta_{t}}-\sin\theta \cos\theta d\theta +\\ & (\frac{R_{C}^{2}}{ES}+\frac{R_{C}}{EA})(\int_{0}^{\theta_{0}}-\sin\theta (\cos\theta -3)d\theta +\int_{\theta_{t}}^{\pi }-\sin\theta (\cos\theta -3)d\theta )+\\ & (\frac{kR_{C}}{GA}-\frac{2R_{C}}{EA})(\int_{0}^{\theta_{0}}\sin\theta \cos\theta d\theta +\int_{\theta_{t}}^{\pi }\sin\theta \cos\theta d\theta )+\\ & (\frac{R_{Cd}^{2}}{E_{d}S_{d}}+\frac{R_{Cd}}{E_{d}A_{d}})\int_{\theta_{0}}^{\theta_{t}}-\sin\theta (\cos\theta -3)d\theta +(\frac{kR_{Cd}}{G_{d}A_{d}}-\frac{2R_{Cd}}{E_{d}A_{d}})\int_{\theta_{0}}^{\theta_{t}}\sin\theta \cos\theta d\theta \end{array}$$

Similarly, other parameters such as the transverse displacement flexibility $K_{HZ}$, longitudinal displacement flexibility $K_{ZZ}$ under longitudinal force, as well as its transverse displacement flexibility $K_{HM}$ and longitudinal displacement flexibility $K_{ZM}$ under bending moment can be obtained. The details can be seen in Supplementary Materials.

### 2.3. Displacement Functions for the Lattice Structure

Four serpentine units are combined into a lattice structure, as shown in Figure 1b, where the upper and lower ends of the structure are constrained by fixed ends, and the left and right ends are subjected to a pair of equal and opposite forces. The total deformation at the two endpoints in Equation (12) is obtained by the superposition principle and the details can be seen in Supplementary Materials.

(12) $$\begin{array}{ll} \Delta_{L} & =\Delta_{lx}-\Delta_{rx}\\ & =(K_{lSHH}+K_{rSHH})(\frac{\sqrt{3}}{4}F_{1}+\frac{1}{8}F)+(-K_{lSHZ}+K_{rSHZ})(\frac{1}{4}F_{1}-\frac{\sqrt{3}}{8}F)+\\ & \;(K_{lSZH}-K_{rSZH})(\frac{3}{4}F_{1}+\frac{\sqrt{3}}{8}F)+(-K_{lSZZ}-K_{rSZZ})(\frac{\sqrt{3}}{4}F_{1}-\frac{3}{8}F)+\\ & \;[\frac{1}{2}(K_{lSHM}-K_{rSHM})+\frac{\sqrt{3}}{2}(K_{lSZM}+K_{rSZM})]M \end{array}$$

F=1 and $\Delta_{L}$ represent the displacement of the lattice structure under a pair of unit loads with equal size and opposite directions, that is, the flexibility $K_{J}$ for the lattice structure.

### 2.4. Deformation Coordination Relationship for the TTEM

In order to control the temperature-induced deformation of the TTEM, it is necessary to study the relationship between the deformation and coverage (the ratio of the curvature of the excitation layer to the frame layer) of the internal serpentine unit and the external lattice structure. To simplify the calculation, it is assumed that the temperature-induced deformation of the serpentine unit in the external lattice is canceled with each other. For tunable thermal expansion metamaterial in Figure 1c, when a force F induced by the thermal expansion in the serpentine unit acts on the lattice structure, it will deform $\Delta_{L}$ as shown in Figure 1c.

(13) $$\Delta_{L}=K_{J}F$$

In the internal serpentine unit, firstly, it expands $\Delta_{T}$ when heated, then shortened $\Delta_{SHH}$ by the lattice structure reaction force $F^{\prime }$, and its total deformation $\Delta_{L}$ is shown in Figure 1e.

(14) $$\Delta_{SHH}=K_{SHH}F^{\prime }$$

where $\Delta_{SHH}=\Delta_{T}-\Delta_{L}$, $F=F^{\prime }$, $K_{J}$ and $K_{SHH}$ represent the displacement flexibility of the lattice structure and the serpentine unit, respectively. Combining Equation (13) with Equation (14) produces the following equation:

(15) $$\frac{\Delta_{L}}{K_{J}}=\frac{\Delta_{T}-\Delta_{L}}{K_{SHH}}$$

From Equation (15), it can be seen that the flexibility of lattice structure is equal to that of serpentine unit when the final deformation of serpentine unit is only half of the temperature-induced deformation, namely, $\Delta_{L}=\Delta_{T}/2$. Thus, the deformation coordination relationship as shown in Equation (16).

(16) $$K_{J}=K_{SHH}$$

## 3. Results and Discussion

### 3.1. Material Preparation

In this study, we choose polymethyl methacrylate (PMMA, $E_{PMMA}=2500\;\mathrm{Mpa},\alpha_{PMMA}$ = $7.0\times 10^{-5}K^{-1}$) as the internal red actuation layer, and polyimide (PI, $E_{PI}=2500\;\mathrm{Mpa},\alpha_{PI}$ = $3.0\times 10^{-5}K^{-1}$) and polylactide (PLA, $E_{PLA}=2500\;\mathrm{Mpa},\alpha_{PLA}=5.0\times 10^{-5}K^{-1}$) as the external yellow frame layer. The above three materials are divided into two groups, in which the first group (PI+PMMA) is abbreviated as G.1 and the second group (PLA+PMMA) is abbreviated as G.2. All of these resin materials have the same mechanical property, such as Young’s modulus *E* = 2500 MPa and Poisson’s ratio *μ* = 0.3.

The printing, heating, and testing equipment are illustrated in Figure 3. The left end of the serpentine unit as well as the upper and lower ends of the tunable thermal expansion metamaterial, are fixed. The heating rate is set to 1.5833 °C/min from 25 °C to 95 °C and a constant temperature is maintained for 1 h.

### 3.2. Deformation Coordination Relationship

**Figure 4:**
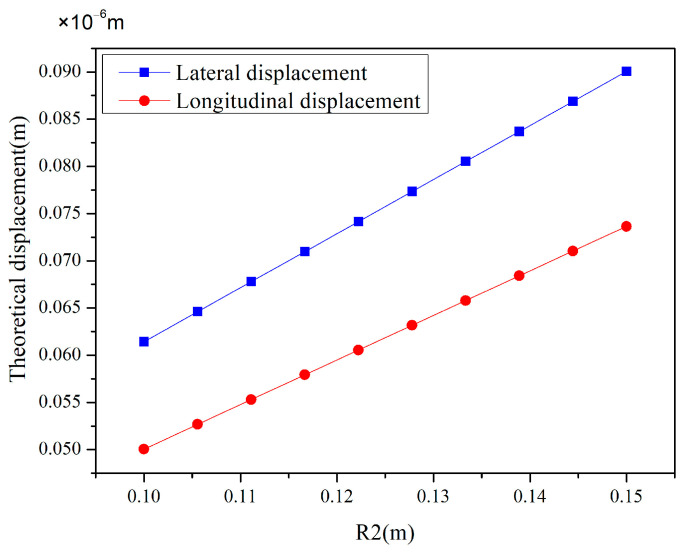
Effect of R_2_ variation on lateral and longitudinal displacements at 50% coverage rate.

Figure 4 clearly depicts the trend of lateral displacement (blue) and longitudinal displacement (red) as the internal radius R2 increases at a fixed coverage rate of 50%. It is evident from the figure that both lateral and longitudinal displacements exhibit a stable linear growth as $R_{2}$ increases. This phenomenon indicates that under the specific conditions of this study, increasing $R_{2}$ leads to an increase in the structural displacement. This linear relationship is crucial for understanding and predicting the behavior of structures under different design parameters.

**Figure 5:**
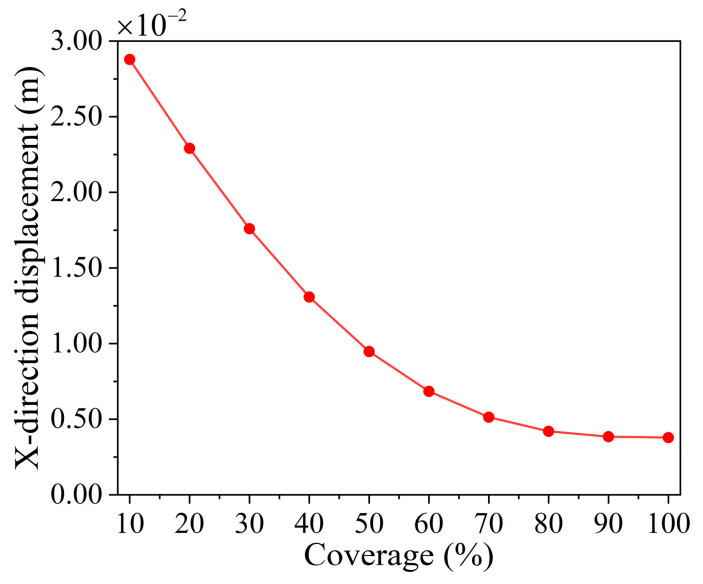
Relationship between lattice structure’s x-direction displacement and coverage rate.

Figure 5 meticulously delineates the functional relationship between the X-direction deformation (in meters) and the coverage rate (in percentage) based on the predictive outcomes of an analytical model. The overall trend of the curve indicates a decrease in the X-direction displacement as the coverage rate increases, providing a clear quantitative basis for understanding the impact of the coverage rate on the deformation behavior of materials or structures.

**Figure 6:**
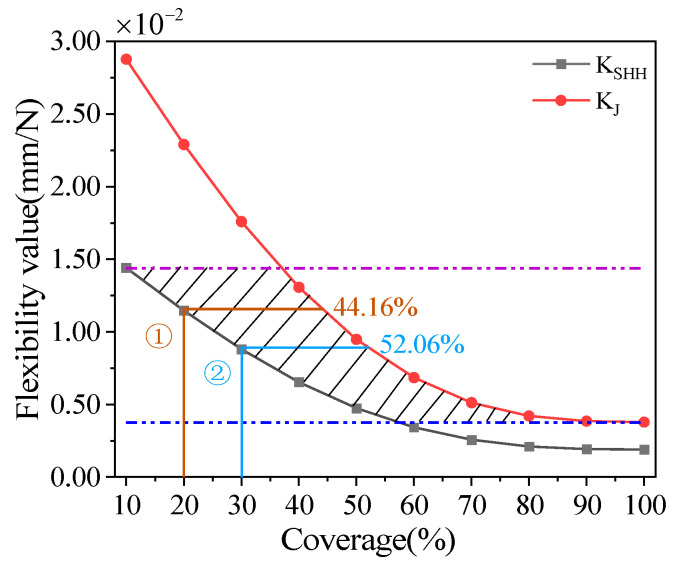
The relationship between flexibility and coverage of the internal serpentine unit and external lattice structure.

Figure 6 shows the design results of the deformation coordination relationship of the tunable thermal expansion metamaterials with a Young’s modulus of 2500 Mpa and a Poisson’s ratio of 0.3. Flexibility analysis for the bi-material serpentine unit and the lattice structure are shown in Supplementary Materials Sections 3 and 4. The feasible region can be determined by the flexibility curve to make the lattice structure’s flexibility $K_{J}$ equal to the flexibility $K_{SHH}$ of the serpentine unit, as shown in the shadow range. When the required compliance of the lattice structure $K_{J}$ and compliance of the serpentine unit $K_{SHH}$ both fall within the feasible range of $0.75\times 10^{-2}\mathrm{mm}/N$ to $1.5\times 10^{-2}\mathrm{mm}/N$, it is necessary to determine the corresponding range of coverage rates. By precisely controlling this range of coverage rates, the desired flexibility can be achieved for both structures, thereby effectively controlling the temperature-induced deformation.

### 3.3. Simulation Verification

**Figure 7:**
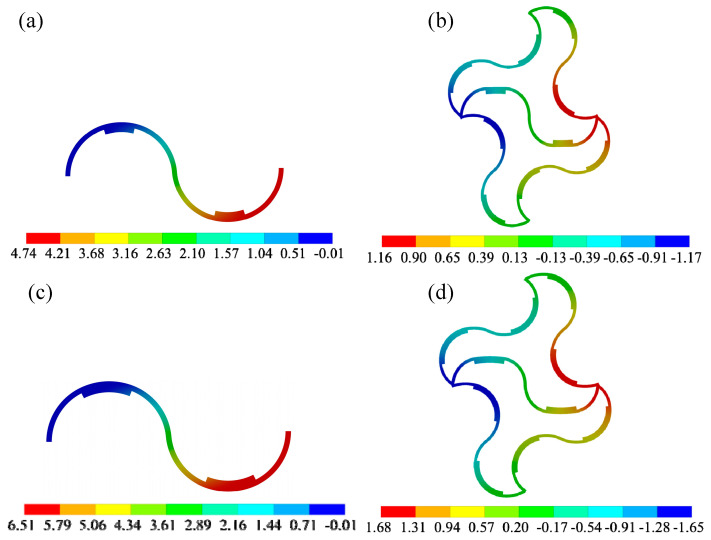
Temperature deformation for the serpentine unit under the coverages of 20% (**a**) and 30% (**b**). The final deformation for the serpentine unit under the coverages of 20% (**c**,**d**).

In order to verify the deformation coordination relationship of the tunable thermal expansion metamaterial, the 2D finite element method is used to analyze two cases, and the specific coverage is shown in Figure 6 ① and ②. The contact surface between the excitation layer and the frame layer adopts a binding method. And the free mesh division with a mesh size of 1 mm is used. The fixed-end constraints are applied to the upper and lower ends of the lattice structure, and the temperature change in the internal serpentine is set between 25 °C and 95 °C. The first group (PI+PMMA) is selected to analyze. For case ①, when the temperature reaches 95 °C, the temperature deformation of the serpentine unit and the final deformation of the left and right ends are 4.74 mm (Figure 7a) and 2.33 mm (Figure 7b), respectively. In the same way, for case ②, the temperature deformation and the final deformation of the serpentine unit are 6.51 mm (Figure 7c) and 3.30 mm (Figure 7d), respectively. Compared with the analytic model, the error of the total deformation is less than 2%.

The remaining sets of validation data are shown in Table 1, where, regardless of G.1 or G.2, the temperature deformation is nearly twice that of the final deformation for the serpentine unit in the metamaterial. In addition, because the interaction between the excitation layer and the framework is ignored after selecting coverage from the shadow range outward, the error gradually increases. This mainly stems from the superposition of errors in the analysis model of the serpentine unit and lattice structure. From Table 1, we can choose a specific coverage rate to achieve better control results. All the simulation results verify that the design method proposed in this paper is basically feasible and can be used before the experiment.

**Figure 8:**
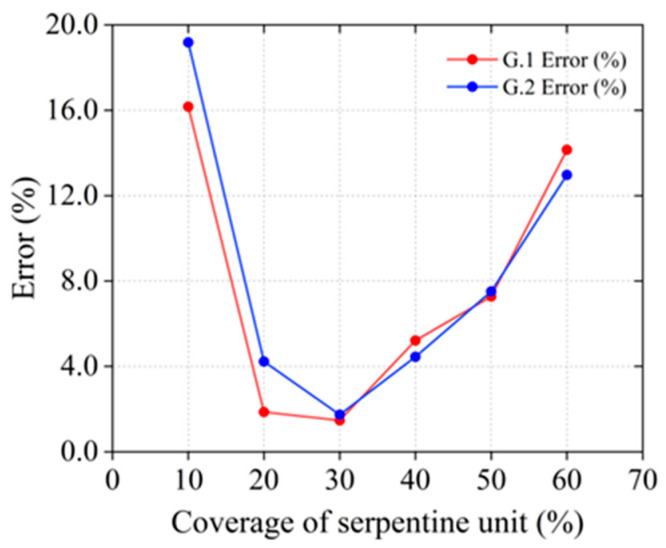
Error comparison between G.1 (PI+PMMA) and G.2 (PLA+PMMA).

Figure 8 provides a comparative analysis of the error percentages for two materials, G.1 (PI+PMMA) and G.2 (PLA+PMMA), across different coverage rates of the serpentine unit. For both materials, the error percentages initially decrease and then increase as the coverage rate of the serpentine unit rises. At a coverage rate of 10%, the error percentage for material G.2 peaks at approximately 19%, while for material G.1, it is around 16% under the same conditions. At a coverage rate of 60%, material G.1 achieves an error percentage of about 14%, whereas material G.2 records an error percentage of approximately 13%. In summary, both materials demonstrate good performance under the same coverage conditions.

### 3.4. Experimental Verification

**Figure 9:**
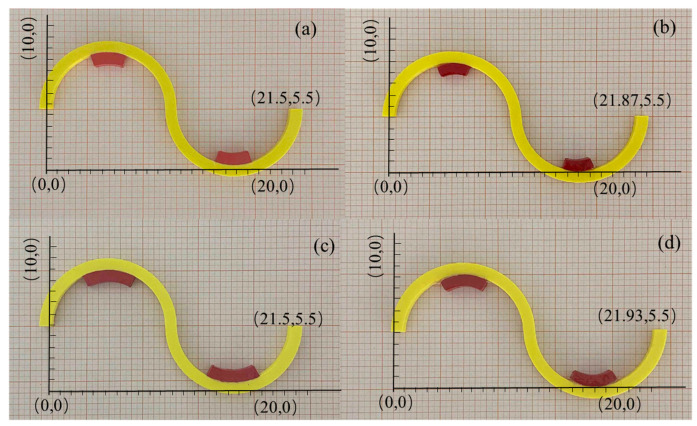
Thermal expansion deformation of the serpentines unit. (**a**) Room temperature (20% coverage); (**b**) after thermal deformation (20% coverage); (**c**) room temperature (30% coverage); (**d**) sample after thermal deformation (30% coverage).

**Figure 10:**
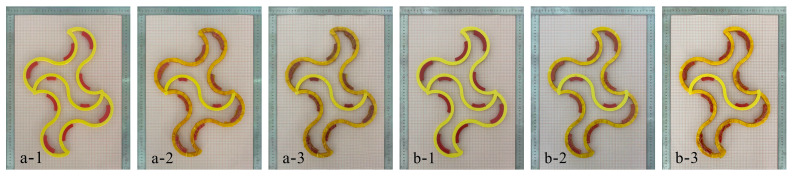
Tunable thermal expansion metamaterial deformation (**a-1**) at room temperature (Case 1); (**a-2**) room temperature+insulation treatment (Case 1); (**a-3**) after thermal deformation (Case 1); (**b-1**) room temperature (Case 2); (**b-2**) room temperature+insulation treatment (Case 2); (**b-3**) after thermal deformation (Case 2).

To avoid measurement errors caused by temperature loss during the measurement process, multiple experiments were conducted on each case and the average measurement value was calculated. Figure 9a,c show the serpentine unit specimens with 20% and 30% coverage at room temperature, respectively. When the temperature changed from 25 °C to 95 °C during the heating process, the temperature deformation was 3.7 mm and 4.3 mm, respectively, as shown in Figure 9b,d. For samples a-2 and b-2 in Figure 10, the total temperature-induced deformation at the left and right ends is 2.1 mm and 2.5 mm in the same temperature range, respectively, as shown in Figure 10a-3,b-3. The reason for the error may come from the loss of temperature and the model difference between the analytical model (2D) and the experiment (3D).

The experimental results indicate that the temperature-induced deformation of the serpentine unit is nearly twice that of the final deformation of the metamaterial, which confirms the temperature-induced deformation coordination relationship proposed in this paper. The finite element method can be employed to calculate the specific deformation dimensions within the feasible region. Analytical and simulation methods can be applied to the structural design of temperature-sensitive bi-metamaterials, and can even be used to precisely adjust parameters in practical engineering to achieve non-deformation in specific directions.

## 4. Conclusions

In this paper, the deformation characteristics of tunable thermal expansion metamaterials are explored through analytical and simulation approaches, leading to the following conclusions:

1. Displacement flexibility functions for the serpentine units and lattice structure within tunable thermal expansion metamaterials are formulated using the principle of virtual work and the method of superposition.
2. It is discovered that the internal serpentine unit and the external lattice structure exhibit equivalent flexibility when the final deformation of the serpentine unit is half of its temperature-induced deformation. Based on this finding, this study presents a method for analyzing the deformation coordination relationship between the lattice and serpentine unit coverage rates in metamaterials.
3. The proposed deformation coordination relationship is validated through finite element analysis and experimental methods. With an internal unit coverage rate of 30% and an external lattice structure coverage rate of 52.06%, the prediction error is maintained within 1.47%. These research outcomes provide a theoretical foundation and design guidance for precise temperature-induced deformation control of bi-material serpentine network structural units.

## Figures and Tables

**Table 1:** Deformation of two groups of the serpentine units in G.1 and G.2.

Coverage of the Serpentine Unit (%)	Lattice Structure Coverage (%)	Temperature Deformation of the Serpentine Unit (mm)		Simulation Value of the Final Deformation of the Serpentine Unit (mm)		Analytic Model of the Final Deformation of the Serpentine Unit (mm)	
		G.1	G.2	G.1	G.2	G.1	G.2
10	36.97	2.93	2.88	1.26	1.16	1.47	1.44
20	44.16	4.74	3.78	2.33	1.81	2.37	1.89
30	52.06	6.51	4.67	3.30	2.29	3.26	2.34
40	61.62	8.18	5.51	4.32	2.85	4.09	2.75
50	74.16	9.62	6.53	5.19	3.35	4.81	3.11
57.24	100	10.60	6.71	4.64	2.92	5.30	3.35

## Data Availability

The original contributions presented in this study are included in the article/supplementary material. Further inquiries can be directed to the corresponding author.

## Supplementary Materials

The following supporting information can be downloaded at: https://www.mdpi.com/article/10.3390/ma18030532/s1, Figure S1. Forces on the lattice structure. Figure S2. Relationship between coverage and flexibility value for the serpentine unit. Figure S3. Relationship between coverage and flexibility value for lattice structure of *K_J__._*
