# Supplementary material for: Analytical Model of Temperature-Induced Deformation for Tunable Thermal Expansion Metamaterial
Source: Materials (Basel). 2025 Jan 24;18(3):532. doi: 10.3390/ma18030532 (PMC11818804; doi:10.3390/ma18030532)
Supplement: Supplementary file 1 [file materials-18-00532-s001.zip › materials-3421625-supplementary.pdf]

Supporting information

1. Displacement function of bi-material serpentine unit

When the serpentine unit is subjected to longitudinal load shown in Figure 3(b), both the transverse and longitudinal displacements of the free end produce. Under the action of longitudinal force F_Z, the internal force equation of each part for the unit can be obtained by the section method.

$$\begin{aligned} &\textit{right section}\text{:}\ \mathrm{M}_1 = F_Z R_C(\cos\theta - 1), F_{S1} = F_Z \sin\theta, F_{N1} = -F_Z \cos\theta \\ &\textit{left section}\text{:}\ \mathrm{M}_2 = F_Z R_C(\cos\theta - 3), F_{S2} = F_Z \sin\theta, F_{N2} = F_Z \cos\theta \end{aligned} \tag{S-1}$$

In order to solve the transverse displacement Δ_{HZ} under the action of unit transverse force, the internal force equation of each part for the unit is obtained by the section method as follows:

$$\begin{aligned} &\textit{right section}\text{:}\ \overline{\mathrm{M}}_1 = R_C \sin\theta, \overline{F}_{S1} = -\cos\theta, \overline{F}_{N1} = -\sin\theta \\ &\textit{left section}\text{:}\ \overline{\mathrm{M}}_2 = -R_C \sin\theta, \overline{F}_{S2} = \cos\theta, \overline{F}_{N2} = -\sin\theta \end{aligned} \tag{S-2}$$

Substituting Eqs. (S-1) and (S-2) into Eq. (4), the transverse displacement function of bi-material serpentine unit under longitudinal force is obtained by section integral method.

$$\Delta_{HZ} = K_{HZ} F_Z \tag{S-3}$$

In order to solve the longitudinal displacement Δ_{ZZ} under the action of unit longitudinal force, the internal force equation of each part for the unit is obtained by the section method in the following.

$$\begin{aligned} &\textit{right section}\text{:}\ \overline{\mathrm{M}}_1 = R_C(\cos\theta - 1), \overline{F}_{S1} = \sin\theta, \overline{F}_{N1} = -\cos\theta \\ &\textit{left section}\text{:}\ \overline{\mathrm{M}}_2 = R_C(\cos\theta - 3), \overline{F}_{S2} = \sin\theta, \overline{F}_{N2} = \cos\theta \end{aligned} \tag{S-4}$$

Substituting Eqs. (S-1) and (S-4) into Eq. (4), the longitudinal displacement function of bi-material serpentine unit under longitudinal force can be obtained by the same method above.

$$\Delta_{ZZ} = K_{ZZ} F_Z \tag{S-5}$$

To sum up, the transverse displacement flexibility K_{HZ} and longitudinal displacement flexibility K_{ZZ} of bi-material serpentine unit under longitudinal force are:

$$K_{HZ}=(\frac{R_C^2}{ES}-\frac{R_C}{EA})(\int_0^{\theta_0}\sin\theta(\cos\theta-1)d\theta+\int_{\theta_t}^{\pi}\sin\theta(\cos\theta-1)d\theta)-$$

$$\begin{aligned}&\frac{kR_C}{GA}(\int_0^{\theta_0}\sin\theta\cos\theta d\theta+\int_{\theta_t}^{\pi}\sin\theta\cos\theta d\theta)+(\frac{R_{Cd}^2}{E_dS_d}-\frac{R_{Cd}}{E_dA_d})\int_{\theta_0}^{\theta_t}\sin\theta(\cos\theta-1)d\theta-\\&\frac{kR_{Cd}}{G_dA_d}\int_{\theta_0}^{\theta_t}\sin\theta\cos\theta d\theta+(\frac{R_C^2}{ES}+\frac{R_C}{EA})(\int_0^{\theta_0}-\sin\theta(\cos\theta-3)d\theta+\\&\int_{\theta_t}^{\pi}-\sin\theta(\cos\theta-3)d\theta)+(\frac{kR_C}{GA}-\frac{2R_C}{EA})(\int_0^{\theta_0}\sin\theta\cos\theta d\theta+\int_{\theta_t}^{\pi}\sin\theta\cos\theta d\theta)+\\&(\frac{R_{Cd}^2}{E_dS_d}+\frac{R_{Cd}}{E_dA_d})\int_{\theta_0}^{\theta_t}-\sin\theta(\cos\theta-3)d\theta+(\frac{kR_{Cd}}{G_dA_d}-\frac{2R_{Cd}}{E_dA_d})\int_{\theta_0}^{\theta_t}\sin\theta\cos\theta d\theta\end{aligned}\tag{S-6}$$

$$\begin{aligned}K_{ZZ}=&\frac{R_C^2}{ES}(\int_0^{\theta_0}(\cos\theta-1)^2d\theta+\int_{\theta_t}^{\pi}(\cos\theta-1)^2d\theta)+\\&\frac{kR_C}{GA}(\int_0^{\theta_0}\sin^2\theta d\theta+\int_{\theta_t}^{\pi}\sin^2\theta d\theta)+\frac{R_C}{EA}(\int_0^{\theta_0}\cos^2\theta d\theta+\int_{\theta_t}^{\pi}\cos^2\theta d\theta)-\\&\frac{2R_C}{EA}(\int_0^{\theta_0}\cos\theta(\cos\theta-1)d\theta+\int_{\theta_t}^{\pi}\cos\theta(\cos\theta-1)d\theta)+\\&\frac{R_{Cd}^2}{E_dS_d}\int_{\theta_0}^{\theta_t}(\cos\theta-1)^2d\theta+\frac{kR_{Cd}}{G_dA_d}\int_{\theta_0}^{\theta_t}\sin^2\theta d\theta+\frac{R_{Cd}}{E_dA_d}\int_{\theta_0}^{\theta_t}\cos^2\theta d\theta-\\&\frac{2R_{Cd}}{E_dA_d}\int_{\theta_0}^{\theta_t}\cos\theta(\cos\theta-1)d\theta+\frac{R_C^2}{ES}(\int_0^{\theta_0}(\cos\theta-3)^2d\theta+\int_{\theta_t}^{\pi}(\cos\theta-3)^2d\theta)+\\&\frac{kR_C}{GA}(\int_0^{\theta_0}\sin^2\theta d\theta+\int_{\theta_t}^{\pi}\sin^2\theta d\theta)+\frac{R_C}{EA}(\int_0^{\theta_0}\cos^2\theta d\theta+\int_{\theta_t}^{\pi}\cos^2\theta d\theta)+\\&\frac{2R_C}{EA}(\int_0^{\theta_0}\cos\theta(\cos\theta-3)d\theta+\int_{\theta_t}^{\pi}\cos\theta(\cos\theta-3)d\theta)+\frac{R_{Cd}^2}{E_dS_d}\int_{\theta_0}^{\theta_t}(\cos\theta-3)^2d\theta+\\&\frac{kR_{Cd}}{G_dA_d}\int_{\theta_0}^{\theta_t}\sin^2\theta d\theta+\frac{R_{Cd}}{E_dA_d}\int_{\theta_0}^{\theta_t}\cos^2\theta d\theta+\frac{2R_{Cd}}{E_dA_d}\int_{\theta_0}^{\theta_t}\cos\theta(\cos\theta-3)d\theta\end{aligned}\tag{S-7}$$

When the serpentine unit is subjected to bending moment M shown in Figure 3(c), both the transverse and longitudinal displacements of the free end produce. Under the action of the bending moment M, the internal force equations of each part for the unit can be obtained by the section method.

$$\begin{aligned}&\textit{right section}\text{:}\ \mathrm{M}_1=M,F_{S1}=0,F_{N1}=0\\&\textit{left section}\text{:}\ \mathrm{M}_2=M,F_{S2}=0,F_{N2}=0\end{aligned}\tag{S-8}$$

In order to solve the transverse displacement Δ_{HM} under the action of the unit transverse force, the internal force equation of each part for the unit is obtained by the section method as follows:

$$\begin{aligned} &right\ section\text{:}\ \ \overline{\mathrm{M}}_1 = R_C \sin\theta, \overline{F}_{S1} = -\cos\theta, \overline{F}_{N1} = -\sin\theta \\ &left\ section\text{:}\ \ \overline{\mathrm{M}}_2 = -R_C \sin\theta, \overline{F}_{S2} = \cos\theta, \overline{F}_{N2} = -\sin\theta \end{aligned} \tag{S-9}$$

Substituting Eqs. (S-8) and (S-9) into Eq. (4), the transverse displacement function of the bi-material serpentine unit under bending moment force is obtained by section integral method.

$$\Delta_{HM} = K_{HM} M \tag{S-10}$$

In order to solve the longitudinal displacement Δ_{ZM} under the action of the unit longitudinal force, the internal force equation of each part for the unit is obtained by the section method in the following:

$$\begin{aligned} &right\ section\text{:}\ \ \overline{\mathrm{M}}_1 = R_C(\cos\theta - 1), \overline{F}_{S1} = \sin\theta, \overline{F}_{N1} = -\cos\theta \\ &left\ section\text{:}\ \ \overline{\mathrm{M}}_2 = R_C(\cos\theta - 3), \overline{F}_{S2} = \sin\theta, \overline{F}_{N2} = \cos\theta \end{aligned} \tag{S-11}$$

Substituting Eqs. (S-8) and (S-11) into Eq. (4), the longitudinal displacement function of the bi-material serpentine unit under transverse force can be obtained by the same method above.

$$\Delta_{ZM} = K_{ZM} M \tag{S-12}$$

To sum up, the transverse displacement flexibility K_{HM} and longitudinal displacement flexibility K_{ZM} of the bi-material serpentine unit under bending moment force are:

$$\begin{aligned} K_{HM} = &\left(\frac{R_C}{ES} - \frac{1}{EA}\right)\left(\int_0^{\theta_0} \sin\theta d\theta + \int_{\theta_t}^{\pi} \sin\theta d\theta\right) + \left(\frac{R_{Cd}}{E_d S_d} - \frac{1}{E_d A_d}\right)\int_{\theta_0}^{\theta_t} \sin\theta d\theta + \\ &\left(-\frac{R_C}{ES} - \frac{1}{EA}\right)\left(\int_0^{\theta_0} \sin\theta d\theta + \int_{\theta_t}^{\pi} \sin\theta d\theta\right) + \left(-\frac{R_{Cd}}{E_d S_d} - \frac{1}{E_d A_d}\right)\int_{\theta_0}^{\theta_t} \sin\theta d\theta \end{aligned} \tag{S-13}$$

$$\begin{aligned} K_{ZM} = &\frac{R_C}{ES}\left(\int_0^{\theta_0} (\cos\theta - 1) d\theta + \int_{\theta_t}^{\pi} (\cos\theta - 1) d\theta\right) + \frac{1}{EA}\left(\int_0^{\theta_0} -\cos\theta d\theta + \int_{\theta_t}^{\pi} -\cos\theta d\theta\right) + \\ &\frac{R_{Cd}}{E_d S_d}\int_{\theta_0}^{\theta_t} (\cos\theta - 1) d\theta + \frac{1}{E_d A_d}\int_{\theta_0}^{\theta_t} -\cos\theta d\theta + \frac{R_C}{ES}\left(\int_0^{\theta_0} (\cos\theta - 3) d\theta + \int_{\theta_t}^{\pi} (\cos\theta - 3) d\theta\right) + \\ &\frac{1}{EA}\left(\int_0^{\theta_0} \cos\theta d\theta + \int_{\theta_t}^{\pi} \cos\theta d\theta\right) + \frac{R_{Cd}}{E_d S_d}\int_{\theta_0}^{\theta_t} (\cos\theta - 3) d\theta + \frac{1}{E_d A_d}\int_{\theta_0}^{\theta_t} \cos\theta d\theta \end{aligned} \tag{S-14}$$

2. Displacement function for the lattice structure

The four serpentine unit structures are combined into a lattice structure. Assuming that the upper and lower ends for the structure are constrained by fixed ends, the left and right ends for the structure are subjected to a pair of equal and opposite forces as shown in Figure S-1(a). By using the section method, the force analysis for the lower half of the lattice structure is performed to solve the flexibility of the structure, as shown in Figure S-1(b).

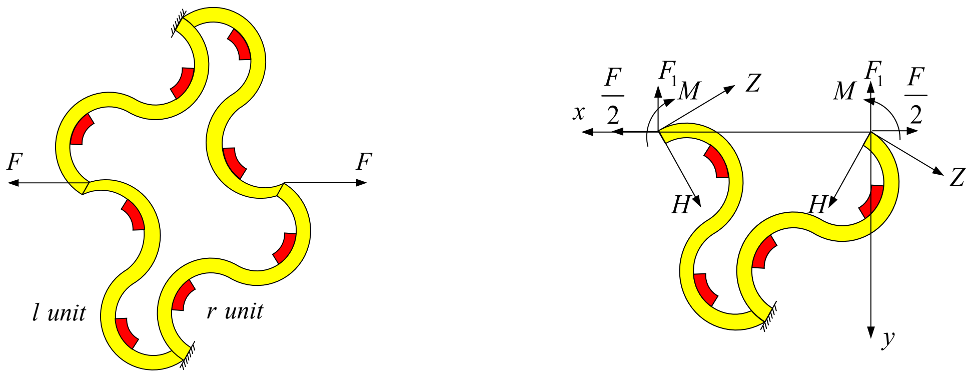

(a) Force analysis for lattice structure

(b) Force analysis of the lower part for the lattice structure

Figure S-1. Forces on the lattice structure

The origin of the *H* and *Z* axes for the serpentine unit is defined to intersect at the outer end point of the free end section, in which the *H*-axis is parallel to the section and points to the fixed end of the unit, and the *Z*-axis is rotated 90° counterclockwise for the *H*-axis. Therefore, the *H*-axis and *Z*-axis of r unit and l unit are shown in Figure S-1(b). It can be seen from this figure that the forces of projection in the *H*-axis and *Z*-axis for r unit are:

$$\begin{aligned} F_H &= -F_1\cos\frac{\pi}{6}-\frac{F}{2}\cos\frac{\pi}{3} \\ F_Z &= \frac{F}{2}\cos\frac{\pi}{6}-F_1\cos\frac{\pi}{3} \end{aligned} \tag{S-15}$$

According to the superposition principle, the projection Δ_{rH} on the *H*-axis and the projection Δ_{rZ} on the *Z*-axis for the free endpoint displacement of r unit caused by the projection force F_H, the projection force F_Z and the bending moment M are:

$$\begin{aligned} \Delta_{rH} &= K_{rSHH}F_H + K_{rSHZ}F_Z + K_{rSHM}M \\ &= K_{rSHH}(-F_1\cos\frac{\pi}{6}-\frac{F}{2}\cos\frac{\pi}{3}) + K_{rSHZ}(\frac{F}{2}\cos\frac{\pi}{6}-F_1\cos\frac{\pi}{3}) + K_{rSHM}M \\ \Delta_{rZ} &= K_{rSZH}F_H + K_{rSZZ}F_Z + K_{rSZM}M \\ &= K_{rSZH}(-F_1\cos\frac{\pi}{6}-\frac{F}{2}\cos\frac{\pi}{3}) + K_{rSZZ}(\frac{F}{2}\cos\frac{\pi}{6}-F_1\cos\frac{\pi}{3}) + K_{rSZM}M \end{aligned} \tag{S-16}$$

where K_{rSHH} and K_{rSZH} are the displacement flexibility along the H-axis and the Z-axis of r serpentine unit under projection force F_H, respectively; K_{rSHZ} and K_{rSZZ} are the displacement flexibility along the *H*-axis and the *Z*-axis of r serpentine unit under projection force F_Z, respectively; K_{rSHM} and K_{rSZM} are the displacement flexibility along the H-axis and the Z-axis of r serpentine unit counterclockwise bending moment M, respectively.

The Δ_{rH} and Δ_{rZ} are projected to the x-axis and y-axis, then the horizontal displacement Δ_{rx} in x-axis and the vertical displacement Δ_{ry} in y-axis for the free endpoint of r unit are:

$$\begin{aligned}\Delta_{rx} &= \Delta_{rH}\cos\frac{\pi}{3} - \Delta_{rZ}\cos\frac{\pi}{6} \\ &= (-\frac{\sqrt{3}}{4}F_1 - \frac{1}{8}F)(K_{rSHH} - \sqrt{3}K_{rSZH}) + (\frac{\sqrt{3}}{8}F - \frac{1}{4}F_1)(K_{rSHZ} - \sqrt{3}K_{rSZZ}) + \\ &\quad \frac{M}{2}(K_{rSHM} - \sqrt{3}K_{rSZM})\end{aligned} \tag{S-17}$$

$$\begin{aligned}\Delta_{ry} &= \Delta_{rH}\cos\frac{\pi}{6} + \Delta_{rZ}\cos\frac{\pi}{3} \\ &= (-\frac{\sqrt{3}}{4}F_1 - \frac{F}{8})(\sqrt{3}K_{rSHH} + K_{rSZH}) + (\frac{\sqrt{3}}{8}F - \frac{F_1}{4})(\sqrt{3}K_{rSHZ} + K_{rSZZ}) + \\ &\quad \frac{M}{2}(\sqrt{3}K_{rSHM} + K_{rSZM})\end{aligned} \tag{S-18}$$

Similarly, the horizontal displacement Δ_{lx} in x-axis and the vertical displacement Δ_{ly} in y-axis for the free endpoint of l unit are:

$$\begin{aligned}\Delta_{lx} &= -\Delta_{lH}\cos\frac{\pi}{3} - \Delta_{lZ}\cos\frac{\pi}{6} \\ &= (\frac{\sqrt{3}}{4}F_1 + \frac{F}{8})(K_{lSHH} + \sqrt{3}K_{lSZH}) + (-\frac{F_1}{4} + \frac{\sqrt{3}}{8}F)(K_{lSHZ} + \sqrt{3}K_{lSZZ}) + \\ &\quad \frac{M}{2}(K_{lSHM} + \sqrt{3}K_{lSZM})\end{aligned} \tag{S-19}$$

$$\begin{aligned}\Delta_{ly} &= \Delta_{lH}\cos\frac{\pi}{6} - \Delta_{lZ}\cos\frac{\pi}{3} \\ &= (-\frac{\sqrt{3}}{4}F_1 - \frac{F}{8})(\sqrt{3}K_{lSHH} - K_{lSZH}) + (\frac{F_1}{4} - \frac{\sqrt{3}}{8}F)(\sqrt{3}K_{lSHZ} - K_{lSZZ}) + \\ &\quad \frac{M}{2}(-\sqrt{3}K_{lSHM} + K_{lSZM})\end{aligned} \tag{S-20}$$

where K_{lSHH} and K_{lSZH} are the displacement flexibility along the *H*-axis and the *Z*-axis of l serpentine unit under projection force F_H, respectively; K_{lSHZ} and K_{lSZZ} are the displacement flexibility along the *H* axis and the *Z* axis of l serpentine unit under projection force F_Z, respectively; K_{lSHM} and K_{lSZM} are the displacement flexibility along the *H* axis and the *Z*-axis of l serpentine unit counterclockwise bending moment M, respectively. Δ_{lH} and Δ_{lZ} represents the displacement of the l element along the *H*-axis and *Z*-axis, respectively.

Assuming that the end points of r unit and l unit only produce horizontal displacement

but not vertical displacement.

$$\Delta_{ly} = 0 \qquad \Delta_{ry} = 0 \tag{S-21}$$

The unknown loads F_1 and M are solved by combining Eqs. (S-18), (S-20) and Eq. (S-21), and then substitute them into Eqs. (S-17) and (S-19) to obtain the horizontal displacement Δ_{rx} for r unit endpoint and the horizontal displacement Δ_{lx} for l unit endpoint. Finally, the total deformation Δ_L at the left and right endpoints is gained for the lattice structure subjected to a pair of equal and opposite forces.

$$\Delta_L = \Delta_{lx} - \Delta_{rx} \tag{S-22}$$

3. Flexibility analysis for the bi-material serpentine unit

In order to verify the results of the flexibility theory for the bi-material serpentine unit, the transverse and longitudinal displacement flexibilities of the serpentine unit under three basic loads are calculated by the finite element method as shown in Figure S-2, where the abscissa represents the coverage of the actuation layer ($r\theta_1 / 180°$). The transverse flexibility K_{HH} and longitudinal flexibility K_{ZH} under transverse force for the serpentine unit decrease with the increase of the actuation layer coverage, while flexibilities K_{HZ} and K_{ZZ} also show the same trend under longitudinal force. The value of the transverse flexibility K_{HM} is close to zero under the bending moment, indicating that there is almost no transverse displacement under the bending moment. The value of its longitudinal flexibility K_{ZM} is negative because the free end of the serpentine unit produces upward longitudinal displacement under counterclockwise bending, and the displacement decreases with the increasement of the coverage of the actuation layer.

Comparing the analytic model and simulated values, the relative errors of the displacement flexibilities K_{HH}, K_{ZH} and K_{HZ} are all within 10 %. Under the longitudinal force, the relative error between analytic model and simulated values of longitudinal displacement flexibility K_{ZZ} increases with the coverage increase. When the coverage is less than or equal to 90 %, the relative error is less than 20 %. When the coverage rate is 100 %, the relative error reaches 27 %. Under bending moment load, the relative error between the analytic model and simulation values of longitudinal displacement flexibility K_{ZM} increases with the coverage rate increases. When the coverage rate is less than or equal to 80 %, the relative error is within 20 %. When

the coverage rate is 100 %, the relative error reaches 41 %. The results show that it is practical and feasible to calculate the flexibility of serpentine unit by using the virtual work principle, which can provide a theoretical basis for the experimental design.

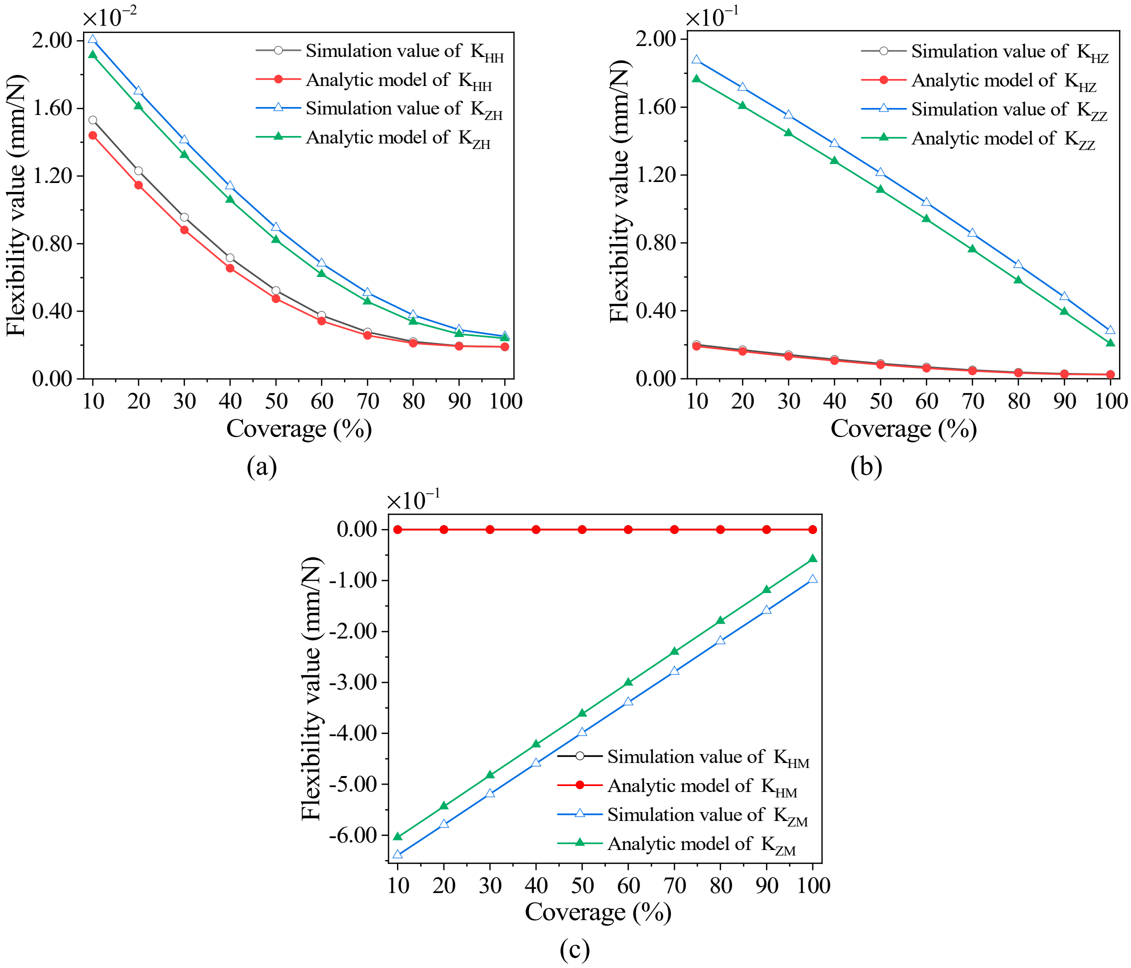

Figure S-2. (a–c) Relationship between coverage and flexibility value for the serpentine unit

4. Flexibility analysis for the lattice structure

Figure S-3 shows the relationship between the flexibility value of the lattice structure and the coverage of the actuation layer. It can be seen from the figure that the flexibility value of the lattice structure decreases with the increase of the coverage of the excitation layer. This is because the existence of the internal actuation layer hinders the deformation of the lattice structure under the force at both ends. It can be seen that the analysis model is in good agreement with the simulation values. The results show that the method of calculating the displacement flexibility of lattice structure by superposition principle is correct and effective.

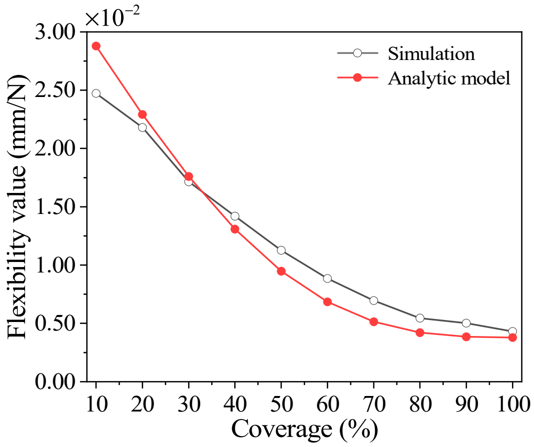

Figure S-3. Relationship between coverage and flexibility value for lattice structure of K_J
